# Hydrogen peroxide and disease: towards a unified system of pathogenesis and therapeutics

**DOI:** 10.1186/s10020-020-00165-3

**Published:** 2020-05-07

**Authors:** Jay Pravda

**Affiliations:** Palm Beach Gardens, Florida USA

**Keywords:** Hydrogen peroxide, Ulcerative colitis, Sepsis, Systemic lupus erythematosus, Glutathione, Redox homeostasis, Redox balance

## Abstract

Although the immune response has a prominent role in the pathophysiology of ulcerative colitis, sepsis, and systemic lupus erythematosus, a primary immune causation has not been established to explain the pathogenesis of these diseases. However, studies have reported significantly elevated levels of colonic epithelial hydrogen peroxide (a known colitic agent) in ulcerative colitis prior to the appearance of colitis. And patients with sepsis are reported to have toxic levels of blood hydrogen peroxide, whose pathologic effects mirror the laboratory and clinical abnormalities observed in sepsis. More recently, evidence supports a causal role for cellular hydrogen peroxide (a potent apoptotic agent) in the enhanced apoptosis believed to be the driving force behind auto-antigenic exposure and chronic immune activation in systemic lupus erythematosus. The different biological properties of hydrogen peroxide exert distinct pathologic effects depending on the site of accumulation within the body resulting in a unique disease patho-phenotype. On a cellular level, the build-up of hydrogen peroxide triggers apoptosis resulting in systemic lupus erythematosus, on a tissue level (colonic epithelium) excess hydrogen peroxide leads to inflammation and ulcerative colitis, and on a systemic level the pathologic effects of toxic concentrations of blood hydrogen peroxide result in bioenergetic failure and microangiopathic dysfunction leading to multiple organ failure and circulatory shock, characteristic of advanced sepsis. The aim of this paper is to provide a unified evidence-based common causal role for hydrogen peroxide in the pathogenesis of ulcerative colitis, sepsis, and systemic lupus erythematosus. Based on this new theory of pathogenesis, a novel evidence-based treatment of sepsis is also discussed.

## Introduction

Hydrogen peroxide (H_2_O_2_) is produced by every cell in the body and has an important physiological role in cellular processes such as membrane signal transduction, gene expression, cell differentiation, insulin metabolism, cell shape determination and growth factor induced signaling cascades (Di Marzo et al. 2018; Lennicke et al. 2015; Sies 2014). However, when produced in excess, cellular H_2_O_2_ has been implicated in the development of disease.

**Fig. 1:**
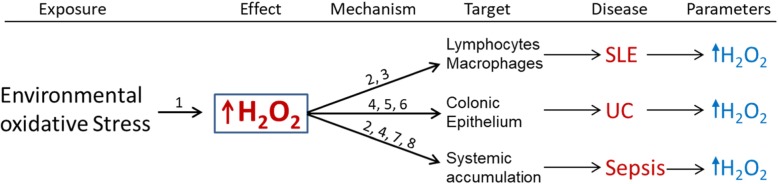
Hydrogen peroxide and disease: A unified mechanism of pathogenesis. Environmental oxidative stress (infections, stress, xenobiotics etc.) leads to increased cellular hydrogen peroxide (H_2_O_2_). Significantly elevated levels of H_2_O_2_ have been documented in the colonic mucosa of patients with ulcerative colitis prior to the appearance of colitis, and toxic levels of H_2_O_2_ have been reported in blood of patients with sepsis. Cumulative evidence also supports a casual role for excess lymphocyte and macrophage H_2_O_2_ in the pathogenesis of systemic lupus erythematosus. H_2_O_2_ has distinct properties that can lead to the development of each disease. They are: **1**) increased by environmental oxidative stress exposure; **2**) Potent apoptotic agent; **3**) Impairment of phagocytosis; **4**) Biomembrane permeability; **5**) Chemotactic for neutrophils; **6**) Oxidant induced intestinal barrier disruption; **7**) Enzyme inhibition and **8**) Hypotensive agent (Pravda 2005; Shenep et al. 1985; Pravda 2019a; Redza-Dutordoir and Averill-Bates 2016; Xiang et al. 2016; Oosting et al. 1990; Möller et al. 2019; Klyubin et al. 1996; Rao et al. 1997; Tatsumi and Kako 1993; Tretter and Adam-Vizi 2000) The resulting disease phenotype is a function of one or more properties of H_2_O_2_ and the target cell in which H_2_O_2_ accumulate; i.e., lymphocytes and macrophages in SLE, colonic epithelium in ulcerative colitis and systemic accumulation in sepsis. The capacity of cellular H_2_O_2_ to increase in response to environmental oxidative stressors is critical to disease development. Determination of specific cell target and subsequent disease phenotype is dependent upon environmental oxidant stress exposure and differential tissue reductive (H_2_O_2_ neutralizing) capacity influenced by genetic or epigenetic predisposition

A causal role for H_2_O_2_ in the pathogenesis of ulcerative colitis has been proposed (Pravda 2005). This is supported by significantly elevated colonic mucosal H_2_O_2_ (a known colitic agent) reported prior to the appearance of colonic inflammation in patients with ulcerative colitis (UC) (Santhanam et al. 2007; Meyer et al. 1981; Sheenan and Brynjolfsson 1960). Cumulative evidence also supports a causal role for H_2_O_2_ in the development of sepsis (Pravda 2014). And toxic levels of blood H_2_O_2_ been documented in patients with sepsis (van Asbeck et al. 1995). H_2_O_2_ toxicity can result in laboratory and clinical abnormalities observed in sepsis, including immunosuppression, bioenergetic organ failure and hypotension among others (Pravda 2014; Shenep et al. 1985). Cumulative evidence likewise supports a causal role for H_2_O_2_ (a potent apoptotic agent) in the amplified lymphocyte and macrophage apoptosis observed in systemic lupus erythematosus (SLE) (Pravda 2019a). Excessive lymphocyte and macrophage apoptosis can lead to enhanced auto-antigenic exposure and chronic auto-immune activation, which is characteristic of SLE (Pravda 2019a). The variety of disease patho-phenotypes exhibited by H_2_O_2_ is made possible by its distinct properties and the target cell/tissue type in which H_2_O_2_ accumulates. Target sites vary from a cellular level in SLE (lymphocytes/macrophage) to a tissue level in UC (colonic epithelium) or systemically in sepsis (Fig. 1). The next section presents the evidence supporting a common causal role for H_2_O_2_ in the in the above three diseases.

## H_2_O_2_ - A common causal role

### Ulcerative colitis

Ulcerative colitis is a major form of inflammatory bowel disease that strikes in the prime of life, mainly in late adolescence to early adulthood (Magro et al. 2017). The rising worldwide incidence of UC has made it a global disease affecting nearly a million Americans (Ng et al. 2017). Ulcerative colitis follows a chronic relapsing and remitting course characterized by abdominal pain, bloody diarrhea, urgency and tenesmus, all of which are related to inflammation of the large intestine (Tripathi and Feuerstein 2019). An immune abnormality is the generally accepted mechanism thought to cause this condition despite extensive research conducted since the mid-twentieth century having failed to establish a primary antecedent immune abnormality in individuals with UC or their family members. (Kirsner 1988; Kirsner 2001). However, significantly elevated levels of H_2_O_2_ have been documented in the colonic epithelium prior to the appearance of inflammation in individuals with UC suggesting a causal role in the development of this disease (Santhanam et al. 2007).

Excess H_2_O_2_ produced by colonocytes (colonic epithelial cells) can easily diffuse through the cell membrane to the extracellular space. H_2_O_2_’s unique properties of cell membrane permeability, long life, potent oxidizing potential and neutrophilic chemotactic ability combine to promote oxidative disintegration of colonic epithelial tight junctional proteins while attracting white blood cells into the colonic epithelium, both of which lead to colonic inflammation and eventual ulcerative colitis (Pravda 2019b). Supporting this interpretation are studies reporting the development of ulcerative colitis in animals and humans after colonic installation of H_2_O_2_ (Meyer et al. 1981; Sheenan and Brynjolfsson 1960). Colonic inflammation analogous to human ulcerative colitis is also reported in glutathione peroxidase knockout mice that are unable to neutralize H_2_O_2_ resulting in colonocyte accumulation of H_2_O_2_ and colitis (Esworthy et al. 2001).

Hydrogen peroxide is produced by several sub-cellular structures whose production of H_2_O_2_ can be increased by environmental factors (called oxidative stressors). The cumulative effect of environmental oxidative stressors can lead to the buildup of colonocyte H_2_O_2_ and eventual UC. For example, serotonin is an enteric neurotransmitter that is normally released by colonic mucosal enterochromaffin cells in order to stimulate enteric nerve terminals to initiate a colonic peristaltic wave (Beattie and Smith 2008). However stress, a known risk factor for UC relapse, can significantly increase colonic contractility (spasm) with the release of significantly greater amounts of serotonin (Bitton et al. 2003; Grace 1954; Almy et al. 1949). The excess serotonin is taken up by colonocytes and metabolized by mono-amine oxidase (EC#1.4.3.4) to H_2_O_2_ (Sturza et al. 2019). Thus, acute severe or prolonged stress can lead to excess colonocyte serotonin metabolism accompanied by H_2_O_2_ accumulation, which can overwhelm colonocyte reductive capacity and contribute to UC relapse. In this regard, studies have shown that serotonin has a critical role in the pathogenesis of experimental colitis (Ghia et al. 2009).

Other risk factors for UC also increase cellular H_2_O_2_. These include high fat diets that contribute to cellular H_2_O_2_ load via peroxisomal beta-oxidation of long-chain fatty acids, which generates large amounts of H_2_O_2_ (Hou et al. 2011; Lismont et al. 2019; Elsner et al. 2011; Antonenkov et al. 2010). Alcohol also contributes to cellular H2O2 accumulation due to cytochrome oxidase CYP2E1 metabolism, which generates H_2_O_2_ (Hoek et al. 2002). Alcohol also inhibits active transport of glutathione into mitochondria where it is needed to neutralize H_2_O_2_ generated by the electron transport chain thus facilitating mitochondrial build-up of H2O2 (Maher 1997; Fernandez-Checa et al. 1997). Studies have shown that high alcohol intake triples the risk of UC relapse (Jowett et al. 2004). Environmental xenobiotic contaminants such as mercury are severe oxidative stressors that increase cellular H_2_O_2_ by inactivation of thiols such as glutathione, which is critical for H_2_O_2_ elimination (Jan et al. 2011; Rubino 2015). Inhalation of minute amounts of mercury vapor can initiate rectal bleeding and relapse of UC (Cummings and Rosenman 2006).

Cigarette smoking cessation is a powerful risk factor for the development of UC (Sands and Compton n.d.; Odes et al. 2001; Pryorham et al. 2003). Tobacco smoking cessation can trigger UC by removing the reported 82% electron transport chain (ETC) inhibition caused by chemicals in tobacco (Pryor et al. 1992). This allows increased ETC metabolism of reducing equivalents (NADH, FADH2) that generates additional H_2_O_2_. Finally, markedly elevated levels of tissue homocysteine have been reported in colonic mucosa of individuals with UC and a meta-analysis found that serum homocysteine levels were significantly higher in UC groups compared to healthy controls (*P* < 0.001) (Morgenstern et al. 2003; Zhong et al. 2019). Homocysteine is a potent oxidative stressor that increases H_2_O_2_ in several ways. H_2_O_2_ is generated during the oxidation of homocysteine to homocystine, and homocysteine increases levels of superoxide dismutase, which converts superoxide to H_2_O_2_ (Friedman et al. 1999; Upchurch Jr et al. 1997; Wilcken et al. 2000). Homocysteine inhibits glutathione peroxidase (GPx) (a critical H_2_O_2_ neutralizing enzyme) activity by 10-fold, and inhibition of GPx was shown to occur at physiologic (9 μmol/L) concentrations of free homocysteine (Upchurch Jr et al. 1997; Chen et al. 2000). Thus, the cumulative effect of endogenous and exogenous H_2_O_2_ inducing environmental oxidative stressors originating from different sources can contribute to colonocyte build-up of H_2_O_2_ and UC.

Perhaps the most perplexing observation regarding the natural history of ulcerative colitis is why the inflammation almost always begins in the rectum? This can now be easily explained with data from studies showing that reductive capacity progressively decreases from proximal to distal areas of the large intestine, with rectal epithelial cells having the least protection against the buildup of H_2_O_2_ (Hoensch et al. 2006). This causes the rectum to be the first location in the large intestine where H_2_O_2_ will accumulate and cause inflammation after exposure to oxidative stress in individuals with ulcerative colitis.

Based on this new pathogenesis of UC, a novel therapy was designed whose goal was focused on the restoration of colonic redox homeostasis by reducing colonic epithelial hydrogen peroxide, which the data implied was the upstream cause of the inflammation. The therapy was offered to patients with refractory ulcerative colitis over a several-year period and the results published as a case report. In 36 patients with moderate to severe refractory disease, histologic remission (complete mucosal healing) was achieved by 85% in an average of 54 days (Pravda et al. 2019).

### Sepsis

Sepsis is a life-threatening condition that is defined as the body’s extreme response to an infection, which can result in multi-organ failure and fatal hemodynamic shock (Centers for Disease Control n.d.). The exact nature of the body’s response leading to sepsis remains unknown. However, cytotoxic levels of blood hydrogen peroxide of over 18x the accepted upper limit of normal have been documented in the blood of individuals with sepsis and septic shock (van Asbeck et al. 1995). Blood H_2_O_2_ is normally between 1 and 5 μM (close to zero) with an accepted upper limit of 30 μM above which general cytotoxicity begins to set in (Forman et al. 2016). However, values up to 558 μM have been documented in the blood of patients with sepsis and septic shock (van Asbeck et al. 1995).

Hypermetabolism is a hallmark of critical illness such as sepsis and nearly all the fuel utilized to power the increased metabolic activity is supplied by ATP. Most cellular ATP is generated via oxidative phosphorylation which produces H_2_O_2_ as a by-product of electron transport chain (ETC) activity. Under normal conditions H_2_O_2_ is efficiently neutralized however, the abrupt global increase of cellular bioenergetic reactions to several times their normal basal state presents the cell with a large surge of hydrogen peroxide that must be eliminated to avoid accumulation and cell death. Prolonged supraphysiological production of hydrogen peroxide generated by ETC hyperactivity during a hypermetabolic state can overwhelm cellular reductive (antioxidant) systems leading to H_2_O_2_ accumulation within tissues and blood. Hydrogen peroxide is a highly toxic membrane-permeable metabolic poison that can cause severe bioenergetic dysfunction and cellular damage if allowed to accumulate. Continued exposure can lead to the collapse of redox homeostasis, organ failure, microvascular dysfunction and fatal septic shock, as discussed below.

Significant depletion of cellular glutathione (principal H_2_O_2_ reducing agent) in lung and skeletal muscle suggests that these organs have become net H_2_O_2_ generators contributing to the rise of blood H_2_O_2_ in sepsis (Pacht et al. 1991; Hammarqvist et al. 1997). The increased systemic production of H_2_O_2_ is reflected in the depletion of whole blood reductive capacity (ability to remove H_2_O_2_) (Lyons et al. 2001). Systemic depletion of reductive capacity portends a grave outcome as was demonstrated in a study that documented significantly decreased erythrocyte glutathione in sepsis non-survivors vs survivors (*P* < 0.0001) (Karapetsa et al. 2013). Furthermore, cellular exposure to H_2_O_2_ can result in metabolic dysfunction that enhances cellular production of H_2_O_2_ (Zorov et al. 2006). This increases the overall H_2_O_2_ load creating a positive feedback loop (vicious cycle) that increases H_2_O_2_ accumulation in the body.

The systemic toxic effects of H_2_O_2_ mirror the laboratory and clinical abnormalities observed in sepsis such as hyperlactatemia. Sepsis associated hyperlactatemia is a strong independent predictor of mortality (Garcia-Alvarez et al. 2014). H_2_O_2_ can increase cellular lactate by interrupting mitochondrial oxidative energy flux, which is needed to maintain the proton motive force that fuels pyruvate import into the mitochondrial matrix (Bender and Martinou 2016).

H_2_O_2_ is reported to inhibit Krebs’ cycle enzymes such as aconitase, alpha-ketoglutarate dehydrogenase and Succinate Dehydrogenase (Tretter and Adam-Vizi 2000; Tretter and Adam-Vizi 2005; Nulton-Persson and Szweda 2001). Diminished Krebs cycle supplied reducing equivalents (NADH, FADH2) can collapse the mitochondrial proton gradient and impair the proton motive force needed for pyruvate translocase in the inner mitochondrial membrane to transport pyruvate into mitochondria in symport with a proton (Bender and Martinou 2016). The end result is increased cytosolic pyruvate and subsequent conversion to lactate with resulting hyperlactatemia. The effect of a dysfunctional Krebs cycle on serum lactate level is observed in the inherited deficiency of alpha-ketoglutarate dehydrogenase, which is associated with severe congenital hyperlactatemia (Bonnefont et al. 1992).

H_2_O_2_ is also reported to inhibit mitochondrial adenine nucleotide transporter and ATP synthase at exposures as low as 10 μM (Tatsumi and Kako 1993). These enzymes are critical for the synthesis of ATP and their inhibition can lead to bioenergetic failure, which is observed in advanced sepsis (Japiassú et al. 2011). In sepsis, lymphocytes are exposed to H_2_O_2_ concentrations of over 500X the 1 μM needed to induce apoptosis (van Asbeck et al. 1995; Antunes and Cadenas 2001). This results in a significant lymphocyte apoptosis (lymphocyte death) resulting in a marked lymphopenia affecting all lymphoid organs in the body including spleen, thymus, intestinal epithelium, lymph nodes and GI tract lymphoid tissue (Exline and Crouser 2008; Hotchkiss et al. 1999; Hotchkiss et al. 2001; Tinsley et al. 2003; Felmet et al. 2005). Thus, H_2_O_2_ toxicity can account for the profound immunosuppression observed in sepsis.

Microangiopathic dysfunction and hypotension are common findings in advanced sepsis (Pravda 2019a). H_2_O_2_ toxicity results in microangiopathic dysfunction and studies have reported hypotension in an animal model after intravenous administration of H_2_O_2_ (Shenep et al. 1985). Consistent with the known pathogenic effects of H_2_O_2_ is a report describing a fatal case of sepsis with multi-organ failure in a previously healthy 37-year-old man after receiving several intravenous infusions of H_2_O_2_ (Wetter and Davis 2006).

Other clinical abnormalities observed in sepsis such as coagulopathy, encephalopathy, erythrocyte rigidity, glutathione depletion, cardiac dysfunction, methemoglobinemia, and mitochondrial dysfunction are also documented adverse effects of H_2_O_2_ (Pravda 2014; Zanotti-Cavazzoni and Hollenberg 2009; Ohashi et al. 1998; Fredriksson et al. 2006; Brealey et al. 2002; Weiss 1982; Evans et al. 1995; Ballinger et al. 1999) all of which contribute to multiple organ failure observed in sepsis.

In summary, a hypermetabolic bio-energetic state in response to a systemic insult will generate supra-physiologic amounts of H2O2 that can overwhelm systemic reductive (anti-oxidant) capacity leading to the accumulation of this toxic metabolic poison in tissues and blood resulting in multi-organ bioenergetic failure and micro-angiopathic dysfunction observed in sepsis.

The evidence-based specific treatment of sepsis is discussed below.

### Systemic lupus erythematosus

Systemic lupus erythematosus (SLE) is a chronic disease characterized by the production of autoreactive antibodies and cytokines that are thought to have a major role in disease activity and progression, which is marked by inflammation and multiple organ damage. The etiology and pathogenesis of SLE remain unknown (Rekvig 2018). However, exposure of the adaptive immune system to intracellular auto-antigens is thought to be a principal mechanism that initiates chronic immune activation giving rise to auto-antibody and cytokine production.

Apoptosis is believed to play a significant role in pathological autoantigen presentation because of the sheer volume of cellular mass normally undergoing apoptosis amounting to 150 billion cells per day or over 10% of total cellular body mass per month (Pravda 2019a; Elliott and Ravichandran 2016). If apoptosis of this large dying cell mass is allowed to proceed unchecked, it would continuously expose intracellular autoantigens to the adaptive immune system as dying apoptotic cells undergo secondary necrosis and intracellular autoantigenic contents are released into the extracellular environment or bloodstream where they would elicit an immune response (Pravda 2019a; Jorge and Means 2019; Caruso and Poon 2018). This does not normally happen because the adaptive immune system is mostly shielded from exposure to autoantigens by phagocytes (i.e. macrophages) that can identify cells undergoing apoptosis and target them for phagocytosis, which safely degrades autoantigens, preventing immune activation (Pravda 2019a; Arandjelovic and Ravichandran 2015; Yoon 2017; Munoz et al. 2008).

However, in SLE apoptosis is increased and phagocytosis is contemporaneously impaired leading to enhanced and prolonged autoantigenic exposure (Yoon 2017; Mistry and Kaplan 2017; Herrmann et al. 1998). In particular, enhanced lymphocyte apoptosis and impaired macrophage phagocytosis observed in patients with SLE are postulated to play a prominent role in the development of this condition (Pravda 2019a). Cumulative evidence supports a causal role for metabolically generated H_2_O_2_ during lymphocyte activation leading to enhanced lymphocyte apoptosis and subsequent auto-antigenic exposure resulting in chronic immune activation chacteristic of SLE (Pravda 2019a). H_2_O_2_ is a potent apoptotic agent and lymphocytes are highly sensitive to H_2_O_2_ induced apoptosis, which can occur at exposures of less than 1 μM (Redza-Dutordoir and Averill-Bates 2016; Xiang et al. 2016; Antunes and Cadenas 2001).

A large amount of H_2_O_2_ is generated during lymphocyte activation as a result of acutely increased metabolic activity. Lymphocyte activation has been described as a metabolic “bomb” during which metabolic activity is greatly upregulated to provide the necessary energy in the form of ATP in order to fuel increased metabolic demands that occur during the clonal-expansion response to infection or receptor signal transduction (Buck et al. 2015). The principal source of cellular hydrogen peroxide is mitochondrial electron transport chain auto-oxidation and associated metabolic enzymes (Wong et al. 2017; Mailloux 2018; Tretter and Adam-Vizi 2004). Additional sources of cellular H_2_O_2_ can be found in the endoplasmic reticulum, peroxisomes, and cytosol (Belikov et al. 2015). All of these H_2_O_2_ site generators contribute to the increased metabolic H_2_O_2_ load during lymphocyte activation.

If H_2_O_2_ generated during lymphocyte activation overwhelms the cell’s reductive (antioxidant) capacity), the subsequent H_2_O_2_ buildup can trigger apoptosis. This interpretation is supported by studies reporting that enhanced apoptosis is associated with depleted glutathione in lymphocytes of patients with SLE, and depleted glutathione levels are significantly associated with worse disease in patients with SLE (*p* < 0.006) (Shah et al. 2013; Tewthanom 2008). Because glutathione is the major reducing agent responsible for the neutralization of cellular H_2_O_2_, a reduction in cellular glutathione will result in elevated cellular H_2_O_2_, which can trigger apoptosis and worsening disease. Elevated H_2_O_2_ also causes impaired macrophage phagocytosis, which prevents elimination of apoptotic cells and enhances auto-sensitization (Oosting et al. 1990).

With a self-repleting cell mass that is 5-fold greater than the liver (2 trillion lymphocytes/360 billion liver cells) (Alberts et al. 2002; Bianconi et al. 2013), lymphocytes are a significant and replenishing source of auto-antigenic exposure if widespread lymphocyte activation triggers H_2_O_2_-induced mass lymphocyte apoptosis. Widespread lymphocyte activation and clonal expansion can occur, for example, due to infection. Additionally, lymphocytes also express receptors for estrogen and adrenergic hormones, which can result in mass lymphocyte activation when lymphocytes are exposed to high concentrations of these hormones after female puberty or emotional stress respectively (Khan and Ansar 2016; Kovats 2015; Segerstrom and Miller 2004; Viswanathan and Dhabhar 2005). All three stimuli (infection, female gender and stress) are known SLE exacerbating factors.

Thus, a pathogenetic picture emerges in which lymphocyte activation and increased macrophage activity (phagocytosis) result in excess metabolically generated H_2_O_2_, which depletes cellular glutathione. This facilitates the intracellular build-up of H_2_O_2_ that triggers apoptosis and impaired phagocytosis in lymphocytes and macrophages respectively, both of which combine to cause repeated, enhanced and prolonged exposure of auto-antigens to the adaptive immune system leading to auto-antibody formation with hyper-cytokinemia and eventual SLE. Treatment of SLE patients with N-acetylcysteine to promote the synthesis of glutathione has been shown to reduce diseases activity, supporting a causal role for H_2_O_2_ in the pathogenesis of SLE (Lai et al. 2012; Garcia et al. 2013).

This data implies that SLE does not start out as an auto-immune disorder but becomes one as a result of continual H_2_O_2_ mediated lymphocyte apoptosis and impaired phagocytosis leading to chronic auto-antigenic exposure, adaptive immune system activation and subsequent development of SLE.

### Treatment

In addition to supportive measures and antibiotic therapy (when indicated for infection), there is a need for effective therapeutic interventions to address the underlying causal process in each of these three diseases. Specifically, there is an urgent need for a specific treatment for sepsis, an often-fatal condition that has defied all attempts at therapeutic intervention. Based on the highly elevated blood hydrogen peroxide levels observed in sepsis patients and cumulative evidence supporting a causal role for this toxic metabolite in sepsis development, it is reasonable to expect that reduction of blood hydrogen peroxide will have a significant therapeutic impact in reducing sepsis mortality and the post-sepsis syndrome.

A therapeutic agent that can effectively neutralize hydrogen peroxide upon contact is sodium thiosulfate (STS). The general chemical reaction for the reduction of hydrogen peroxide with sodium thiosulfate yields sodium trithionate, sodium sulfate and water.

## 2 Na_2_S_2_O_3_ + 4 H_2_O_2_ → Na_2_S_3_O_6_ + Na_2_SO_4_ + 4H_2_O

Depending on the relative concentration of reactants, other STS oxidation products are possible including sodium dithionate (Na_2_S2O6), sodium tetrathionate (Na2S4O6) and H_2_SO_4_ (sulfuric acid). STS is approved for use in cyanide poisoning with a recommended dose of 12.5 g over slow IV infusion (10 to 20 min) in adults and 250 mg/kg in children (US Department of Health and Human Services n.d.). Similar dosing regimens can be considered in sepsis. STS is accepted therapy for the treatment cisplatin toxicity (Tsang et al. 2009), calciphylaxis due to chronic renal failure (Nigwekar et al. 2018), and is reported to lower the incidence of cisplatin-induced ototoxic hearing loss in children with hepatoblastoma (Brock et al. 2018). STS is generally well tolerated.

Because blood H_2_O_2_ is significantly elevated in sepsis, the bidirectionality of H_2_O_2_ cell-membrane permeability implies a similar H_2_O_2_ load within the intracellular compartment (van Asbeck et al. 1995). Due to its significant reductive capacity derived from reduced thiols in serum albumin and erythrocyte glutathione, whole blood normally functions as a physiological redox sink for H_2_O_2_ diffusing from the intracellular compartment into the systemic circulation (Roche et al. 2008; Cha and Kim 1996; Tozzi-Ciancarelli et al. 1990). Consequently, systemically elevated H_2_O_2_ indicates depletion of reductive capacity in whole blood and within the intracellular compartment (Lyons et al. 2001).

Thus, the goal of treatment is to reduce blood H_2_O_2_ to normal (less than 30uM) to allow intracellular H_2_O_2_ to diffuse down its concentration gradient into the systemic circulation where it can be neutralized by STS. This suggests that repeat dosing of STS may be necessary as blood H_2_O_2_ is removed by STS, and intracellular H_2_O_2_ re-equilibrates with the intravascular compartment after initial treatment. STS is also reported to replenish intracellular glutathione, which will aid in removal of intracellular H_2_O_2_ and restoration of redox homeostasis (Enongene et al. 2000; Hayden et al. 2005). A decreasing trend in elevated serum lactate indicates that H_2_O_2_-induced Krebs cycle inhibition is being reversed as excess mitochondrial H_2_O_2_ is removed followed by restoration of mitochondrial proton motive force needed to transport pyruvate into the mitochondrial matrix. Decreasing cytosolic pyruvate will enhance the conversion of lactate to pyruvate (in the cytosol) and reduce diffusion of cellular lactate into the systemic circulation contributing to the reduction of serum lactate.

Restoration of vascular responsiveness by STS may cause extant vasoactive measures such as fluid loading or vasopressor therapy to have an unanticipated and deleterious augmented effect. Thus, STS should be administered in the appropriate IV fluids accompanied by continuous monitoring. Finally, if H_2_O_2_ reduction therapy proves to be successful in the treatment of sepsis, we should consider this type of therapy to restore depleted blood reducing equivalents with STS before blood H_2_O_2_ becomes toxically elevated in critically ill individuals.

## Discussion

In each disease, SLE, UC and sepsis, the immune response plays a prominent role in pathophysiology. However, there is no evidence that the immune system is involved in the pathogenesis of these diseases. What starts the colitis in individuals with UC? Why does sepsis cause organ failure and hypotension? And why do individuals with SLE produce auto reactive antibodies against their own DNA? All of these observations can be explained by the multi-faceted effects of hydrogen peroxide.

Therapy aimed at the immune response in UC and SLE does not prevent life-long relapse. And all therapeutic attempts to modulate the immune response in sepsis have failed to improve survival in individuals with this condition. In contrast, hydrogen peroxide provides an evidence-based therapeutic and druggable target for each disease. Colonic mucosal hydrogen peroxide (a known colitic agent) is significantly elevated prior to the appearance of colitis in patients with UC, satisfying the temporal requirement for causality. Similarly, toxic levels of hydrogen peroxide have been reported in the blood of individuals with sepsis, and systemic hydrogen peroxide toxicity mirrors the laboratory and clinical abnormalities observed in patients with sepsis.

Likewise, the evidence strongly suggests a causal role for hydrogen peroxide in the systemic lymphocyte apoptosis observed in individuals SLE leading to auto-antigenic exposure and subsequent auto-antibody formation by the adaptive immune system.

In UC, reduction of colonic hydrogen peroxide led to complete mucosal healing in 85% of 36 patients with refractory disease. In a similar fashion, reduction of toxically elevated blood hydrogen peroxide in sepsis is anticipated to significantly reduce the high mortality and post-discharge morbidity caused by this condition. And based on data implicating a causal role for lymphocyte hydrogen peroxide in SLE, maintenance of normal cellular hydrogen peroxide levels is expected to prevent the apoptosis that leads to auto-antigenic exposure and relapse in this illness.

These three diseases represent the prototypical expression of impaired redox homeostasis leading to excess hydrogen peroxide production. Redox homeostasis is impaired on a cellular (lymphocyte, macrophage) level in SLE, on a tissue level (colonic epithelium) in UC, and in sepsis, redox homeostasis is systemically impaired. The end result of impaired redox homeostasis is a build-up of free (un-neutralized) cellular hydrogen peroxide, which can lead to a different patho-phenotype depending upon the location in the body where it accumulates. Impaired redox homeostasis may also be a causal factor in the pathogenesis of other diseases as well.

The healthcare cost savings that can be achieved by a common therapeutic platform for all three diseases are considerable. Sepsis affects 1.7 million Americans each year with an average inpatient healthcare cost of $18,000 (Rhee et al. 2019; Paoli et al. 2018). Overall, one-sixth develop persistent physical disability or cognitive impairment with healthcare costs to individual patients and families of approximately $50,000 annually (Prescott and Angus 2018; Hajj et al. 2018). This exceeds $44 billion dollars annually for sepsis related healthcare. Annual total costs for the approximately one million individuals with UC are about $12 billion dollars while mean total annual cost for the estimated 320,000 individuals with SLE in the United States is close to $6.5 billion dollars (Pilon et al. 2019; Gandhi et al. 2013; Panopalis et al. 2008). With total expenses for these three diseases exceeding $60 billion dollars a year the beneficial economic ramifications of a common therapeutic approach are substantial.

Redox medicine is still in its infancy and there is much research and work to be done in order to fully understand the range of clinical manifestations of impaired redox homeostasis and its proper treatment. This applies to both oxidative and reductive stress as causal and contributory factors in the pathogenesis and pathophysiology of disease. Because of the continuous metabolic production of cellular hydrogen peroxide, redox homeostasis is intimately associated with cellular metabolism and bioenergetics, which in turn are influenced by genetic predisposition and environmental factors.

Thus, redox medicine can make a significant contribution to understanding how and why we develop disease on a personal and populational level, which can improve individual patient care and lead to new and effective public health initiatives. For example, the slow rise in auto-antibodies years prior to the development of clinical SLE suggests a progressive compromise in cellular redox homeostasis (decreased glutathione) during which time therapeutic intervention to restore redox homeostasis may prevent disease. Likewise, the knowledge that disease can be initiated by impaired redox homeostasis and elevated hydrogen peroxide suggests that the addition of reducing equivalents to the food supply may decrease the rising incidence of redox mediated disease.

## Conclusion

Although immune activation is involved in the pathophysiology of sepsis, ulcerative colitis (UC) and systemic lupus erythematosus (SLE), there is no evidence that immune based mechanisms are responsible for the pathogenesis of these conditions. However, hydrogen peroxide can explain the development of each disease. Hydrogen peroxide is a unique molecule having diverse properties that can manifest as different diseases depending upon the site in the body that is exposed to excessive levels of this toxic metabolite. Hydrogen peroxide is a known colitic agent, and significantly elevated levels of this toxic metabolite have been documented to precede the appearance of colonic inflammation in individuals with ulcerative colitis. Similarly, significantly elevated levels of blood hydrogen peroxide have been reported in patients with sepsis and the systemic toxic effects of hydrogen peroxide mirror the clinical and laboratory abnormalities observed in this often-fatal condition. Finally, evidence suggests that hydrogen peroxide is responsible for enhanced lymphocyte apoptosis and impaired phagocytosis observed in SLE, which ultimately leads to auto-antigenic exposure and chronic immune activation. Although we are all expose to environmental oxidative stress, a subset of individuals is environmentally selected to develop disease as a result of a predisposing genetic makeup encoding for a diminished reductive capacity that facilitates the buildup of hydrogen peroxide in different parts of the body. In each case, the immune system is doing what its normally programmed to do given the circumstances it finds itself in.

The recognition of a causal role for hydrogen peroxide facilitates the rational design of therapeutic intervention based on a common mechanism of disease. The data indicate that in UC and SLE the immune reaction is in response to excess hydrogen peroxide in the colonic epithelium and lymphocytes respectively. This suggests that the appropriate therapy for treatment and long-term remission is reduction of hydrogen peroxide and replenishment of depleted reducing equivalents. Targeted therapy to reduce colonic hydrogen peroxide has shown to be highly effective in reversing colonic inflammation in patients with refractory UC. In a similar fashion, reduction of disease activity has been reported in patients with SLE receiving N-acetylcysteine, a precursor to glutathione, which has a critical role in the elimination cellular hydrogen peroxide in order to prevent hydrogen peroxide induced lymphocyte apoptosis, auto-antigenic exposure and subsequent immune activation. Short term immunosuppression has a role in active UC and SLE in order to mitigate the immune response however, there is no role for immunosuppression in sepsis because the immune system is already severely impaired due to profound lymphoid apoptosis as a result of systemic exposure to toxic levels of hydrogen peroxide. Reduction of elevated blood hydrogen peroxide levels is paramount in sepsis in order to prevent bioenergetic organ failure and hypotension, both of which are toxic effects of hydrogen peroxide exposure. Further studies are needed to explore this novel therapeutic mechanism.

## Data Availability

Not applicable.

## Abbreviations

H_2_O_2_: Hydrogen peroxide
UC: Ulcerative colitis
SLE: Systemic lupus erythematosus
STS: sodium thiosulfate

## References

[CR1] Alberts BAJ, Lewis J, Raff M, Roberts K, Walters P (2002). Molecular biology of the cell.

[CR2] Almy TP, MD, Kern F, Tulin M (1949). Alterations in colonic function in man under stress II. Experimental Production of Sigmoid Spasm in Healthy Persons. Gastroenterology.

[CR3] Antonenkov VD, Grunau S, Ohlmeier S, Hiltunen JK (2010). Peroxisomes are oxidative organelles. Antioxidants Redox Signal.

[CR4] Antunes F, Cadenas E (2001). Cellular titration of apoptosis with steady state concentrations of H_2_O_2_: submicromolar levels of H_2_O_2_ induce apoptosis through Fenton chemistry independent of the cellular thiol state. Free Radic Biol Med.

[CR5] Arandjelovic S, Ravichandran KS (2015). Phagocytosis of apoptotic cells in homeostasis. Nat Immunol.

[CR6] Ballinger SW, Van Houten B, Jin GF (1999). Hydrogen peroxide causes significant mitochondrial DNA damage in human RPE cells. Exp Eye Res.

[CR7] Beattie DT, Smith JA (2008). Serotonin pharmacology in the gastrointestinal tract: a review. Naunyn Schmiedeberg's Arch Pharmacol.

[CR8] Belikov AV, Schraven B, Simeoni L (2015). T cells and reactive oxygen species. J Biomed Sci.

[CR9] Bender T, Martinou JC (2016). The mitochondrial pyruvate carrier in health and disease: to carry or not to carry?. Biochim Biophys Acta.

[CR10] Bianconi E, Piovesan A, Facchin F, Beraudi A, Casadei R, Frabetti F (2013). An estimation of the number of cells in the human body. Ann Hum Biol.

[CR11] Bitton A, Sewitch MJ, Peppercorn MA, Edwardes MD, Shah S, Ransil B (2003). Psychosocial determinants of relapse in ulcerative colitis: a longitudinal study. Am J Gastroenterol.

[CR12] Bonnefont JP, Chretien D, Rustin P, Robinson B, Vassault A, Aupetit J (1992). Alpha-ketoglutarate dehydrogenase deficiency presenting as congenital lactic acidosis. J Pediatr.

[CR13] Brealey D, Brand M, Hargreaves I, Heales S, Land J, Smolenski R (2002). Association between mitochondrial dysfunction and severity and outcome of septic shock. Lancet.

[CR14] Brock PR, Maibach R, Childs M, Rajput K, Roebuck D, Sullivan MJ (2018). Sodium thiosulfate for protection from cisplatin-induced hearing loss. N Engl J Med.

[CR15] Buck MD, O’sullivan D, Pearce EL (2015). T cell metabolism drives immunity. J Exp Med.

[CR16] Caruso S, Poon IK (2018). Apoptotic cell-derived extracellular vesicles: more than just debris. Front Immunol.

[CR17] Centers for Disease Control n.d. What is Sepsis? https://www.cdc.gov/sepsis/what-is-sepsis.html. Accessed 20 Mar 2020.

[CR18] Cha MK, Kim IH (1996). Glutathione-linked thiol peroxidase activity of human serum albumin: a possible antioxidant role of serum albumin in blood plasma. Biochem Biophys Res Commun.

[CR19] Chen N, Liu Y, Greiner CD, Holtzman JL (2000). Physiologic concentrations of homocysteine inhibit the human plasma GSH peroxidase that reduces organic hydroperoxides. J Lab Clin Med.

[CR20] Cummings CE, Rosenman KD (2006). Ulcerative colitis reactivation after mercury vapor inhalation. Am J Ind Med.

[CR21] Di Marzo N, Chisci E, Giovannoni R (2018). The role of hydrogen peroxide in redox-dependent signaling: homeostatic and pathological responses in mammalian cells. Cells..

[CR22] Elliott MR, Ravichandran KS (2016). The dynamics of apoptotic cell clearance. Dev Cell.

[CR23] Elsner M, Gehrmann W, Lenzen S (2011). Peroxisome-generated hydrogen peroxide as important mediator of lipotoxicity in insulin-producing cells. Diabetes..

[CR24] Enongene EN, Sun PN, Mehta CS (2000). Sodium thiosulfate protects against acrylonitrile-induced elevation of glial fibrillary acidic protein levels by replenishing glutathione. Environ Toxicol Pharmacol.

[CR25] Esworthy RS, Aranda R, Martin MG, Doroshow JH, Binder SW, Chu FF (2001). Mice with combined disruption of Gpx1 and Gpx2 genes have colitis. Am J Physiol Gastrointest Liver Physiol.

[CR26] Evans T, Jin H, Elkins N, Shapiro J (1995). Effect of acidosis on hydrogen peroxide injury to the isolated perfused rat heart. Am J Phys.

[CR27] Exline MC, Crouser ED (2008). Mitochondrial mechanisms of sepsis-induced organ failure. Front Biosci.

[CR28] Felmet KA, Hall MW, Clark RS, Jaffe R, Carcillo JA (2005). Prolonged lymphopenia, lymphoid depletion, and hypoprolactinemia in children with nosocomial sepsis and multiple organ failure. J Immunol.

[CR29] Fernandez-Checa JC, Kaplowitz N, Garcia-Ruiz C, Colell A, Miranda M, Mari M, Ardite E, Morales A (1997). GSH transport in mitochondria: defense against TNF-induced oxidative stress and alcohol-induced defect. Am J Phys.

[CR30] Forman HJ, Bernardo A, Davies KJ (2016). What is the concentration of hydrogen peroxide in blood and plasma?. Arch Biochem Biophys.

[CR31] Fredriksson K, Hammarqvist F, Strigard K, Hultenby K, Ljungqvist O, Wernerman J (2006). Derangements in mitochondrial metabolism in intercostal and leg muscle of critically ill patients with sepsis-induced multiple organ failure. Am J Physiol Endocrinol Metab.

[CR32] Friedman G, Goldschmidt N, Friedlander Y, Ben-Yehuda A, Selhub J, Babaey S (1999). A common mutation A1298C in human methylenetetrahydrofolate reductase gene: association with plasma total homocysteine and folate concentrations. J Nutr.

[CR33] Gandhi L, Alemao E, Kawabata H, Hilson J (2013). Prevalence of systemic lupus erythematosus and lupus nephritis in the United States: analysis of commercial and public insurance billing data. Arthritis Rheum.

[CR34] Garcia RJ, Francis L, Dawood M, Lai ZW, S. Faraone SV, and Perl A. Brief report: attention deficit and hyperactivity disorder scores are elevated and respond to N-acetylcysteine treatment in patients with systemic lupus erythematosus. Arthritis and Rheumatism. 2013;651313–18. 10.1002/art.37893 PMC4034122 23400548

[CR35] Garcia-Alvarez M, Marik P, Bellomo R. Sepsis-associated hyperlactatemia. Crit Care. 2014;18(5):503. 10.1186/s13054-014-0503-3 PMC4421917 25394679

[CR36] Ghia JE, Li N, Wang H, Collins M, Deng Y, El-Sharkawy RT, Côté F, Mallet J, Khan WI (2009). Serotonin has a key role in pathogenesis of experimental colitis. Gastroenterology..

[CR37] Grace WJ (1954). Life stress and chronic ulcerative colitis. Ann N Y Acad Sci.

[CR38] Hajj J, Blaine N, Salavaci J, Jacoby D (2018). The “centrality of Sepsis”: A review on incidence, mortality, and cost of care. Healthcare.

[CR39] Hammarqvist F, Luo JL, Cotgreave IA, Andersson K, Wernerman J (1997). Skeletal muscle glutathione is depleted in critically ill patients. Crit Care Med.

[CR40] Hayden MR, Tyagi SC, Kolb L, Sowers JR, Khanna R (2005). Vascular ossification–calcification in metabolic syndrome, type 2 diabetes mellitus, chronic kidney disease, and calciphylaxis–calcific uremic arteriolopathy: the emerging role of sodium thiosulfate. Cardiovasc Diabetol.

[CR41] Herrmann M, Voll RE, Zoller OM, Hagenhofer M, Ponner BB, Kalden JR (1998). Impaired phagocytosis of apoptotic cell material by monocyte-derived macrophages from patients with systemic lupus erythematosus. Arthritis Rheumatism.

[CR42] Hoek JB, Cahill A, Pastorino JG (2002). Alcohol and mitochondria: A dysfunctional relationship. Gastroenterology..

[CR43] Hoensch H, Peters WH, Roelofs HM, Kirch W (2006). Expression of the glutathione enzyme system of human colon mucosa by localisation, gender and age. Curr Med Res Opin.

[CR44] Hotchkiss RS, Swanson PE, Freeman BD, Tinsley KW, Cobb JP, Matuschak GM (1999). Apoptotic cell death in patients with sepsis, shock, and multiple organ dysfunction. Crit Care Med.

[CR45] Hotchkiss RS, Tinsley KW, Swanson PE (2001). Sepsis-induced apoptosis causes progressive profound depletion of B and CD4+ T lymphocytes in humans. J Immunol.

[CR46] Hou JK, Abraham B, El-Serag H (2011). Dietary intake and risk of developing inflammatory bowel disease: a systematic review of the literature. Am J Gastroenterol.

[CR47] Jan AT, Ali A, Haq Q (2011). Glutathione as an antioxidant in inorganic mercury induced nephrotoxicity. J Postgrad Med.

[CR48] Japiassú AM, Santiago AP, Joana da Costa P, Garcia-Souza LF, Galina A, Faria-Neto HC (2011). Bioenergetic failure of human peripheral blood monocytes in patients with septic shock is mediated by reduced F1Fo adenosine-5′-triphosphate synthase activity. Crit Care Med.

[CR49] Jorge AM, Means TK. Abnormalities in immune complex clearance and apoptotic cell clearance. In Dubois’ lupus Erythematosus and related syndromes, D. Wallace D, Hannahs Hahn B. Eds. Philadelphia: Elesevier Inc.; 2019. p. 216–23.

[CR50] Jowett SL, Seal CJ, Pearce MS, Phillips E, Gregory W, Barton JR, Welfare MR (2004). Influence of dietary factors on the clinical course of ulcerative colitis: a prospective cohort study. Gut..

[CR51] Karapetsa M, Pitsika M, Goutzourelas N (2013). Oxidative status in ICU patients with septic shock. Food Chem Toxicol.

[CR52] Khan D, Ansar AS (2016). The immune system is a natural target for estrogen action: opposing effects of estrogen in two prototypical autoimmune diseases. Front Immunol.

[CR53] Kirsner JB (1988). Historical aspects of inflammatory bowel disease. J Clin Gastroenterol.

[CR54] Kirsner JB (2001). Historical origins of current IBD concepts. World J Gastroenterol.

[CR55] Klyubin IV, Kirpichnikova KM, Gamaley IA (1996). Hydrogen peroxide induced chemotaxis of mouse peritoneal neutrophils. Eur J Cell Biol.

[CR56] Kovats S (2015). Estrogen receptors regulate innate immune cells and signaling pathways. Cell Immunol.

[CR57] Lai ZW, Hanczko R, Bonilla E, Caza TN, Clair B, Bartos A (2012). N-acetylcysteine reduces disease activity by blocking mammalian target of rapamycin in T cells from systemic lupus erythematosus patients: A randomized, double-blind, placebo-controlled trial. Arthritis Rheum.

[CR58] Lennicke C, Rahn J, Lichtenfels R, Wessjohann LA, Seliger B (2015). Hydrogen peroxide–production, fate and role in redox signaling of tumor cells. Cell Commun Signal.

[CR59] Lismont C, Revenco I, Fransen M (2019). Peroxisomal hydrogen peroxide metabolism and signaling in health and disease. Int J Mol Sci.

[CR60] Lyons J, Rauh-Pfeiffer A, Ming-Yu Y (2001). Cysteine metabolism and whole blood glutathione synthesis in septic pediatric patients. Crit Care Med.

[CR61] Magro F, Gionchetti P, Eliakim R, Ardizzone S, Armuzzi A, Barreiro-de Acosta M (2017). Third European evidence-based consensus on diagnosis and management of ulcerative colitis. Part 1: definitions, diagnosis, extra-intestinal manifestations, pregnancy, cancer, surveillance, surgery, and ileo-anal pouch disorders. J Crohn's Colitis.

[CR62] Maher J (1997). Exploring alcohol’s effects on liver function. Alcohol Health Res World.

[CR63] Mailloux RJ. Mitochondrial antioxidants and the maintenance of cellular hydrogen peroxide levels. Oxidative Med Cell Longev. 2018;2018. 10.1155/2018/7857251 PMC6051038 30057684

[CR64] Meyer CT, Brand M, DeLuca VA, Spiro HM (1981). Hydrogen peroxide colitis: a report of three patients. J Clin Gastroenterol.

[CR65] Mistry P, Kaplan MJ (2017). Cell death in the pathogenesis of systemic lupus erythematosus and lupus nephritis. Clin Immunol.

[CR66] Möller M.N., Cuevasanta E., Orrico F., Lopez A.C., Thomson L., Denicola A. Diffusion and transport of reactive species across cell membranes. In: (2019) Trostchansky A., Rubbo H. (eds) Bioactive Lipids in Health and Disease. Advances in Experimental Medicine and Biology*.* 1127:3-19. Springer, Cham. 10.1007/978-3-030-11488-6_1 31140168

[CR67] Morgenstern I, Raijmakers MT, Peters WH, Hoensch H, Kirch W (2003). Homocysteine, cysteine, and glutathione in human colonic mucosa. Dig Dis Sci.

[CR68] Munoz LE, van Bavel CC, Franz S, Berden J, Herrmann M, Van Der Vlag J (2008). Apoptosis in the pathogenesis of systemic lupus erythematosus. Lupus..

[CR69] Ng SC, Shi HY, Hamidi N, Underwood FE, Tang W, Benchimol EI (2017). Worldwide incidence and prevalence of inflammatory bowel disease in the 21st century: a systematic review of population-based studies. Lancet..

[CR70] Nigwekar SU, Thadhani R, Brandenburg VM (2018). Calciphylaxis. N Engl J Med.

[CR71] Nulton-Persson AC, Szweda L (2001). Modulation of mitochondrial function by hydrogen peroxide. J Biol Chem.

[CR72] Odes HS, Fich A, Reif S, Halak A, Lavy A, Keter D (2001). Effects of current cigarette smoking on clinical course of Crohn’s disease and ulcerative colitis. Dig Dis Sci.

[CR73] Ohashi K, Yukioka H, Hayashi M, Asada A (1998). Elevated methemoglobin in patients with sepsis. Acta Anaesthesiol Scand.

[CR74] Oosting RS, Van Bree L, Van Iwaarden JF, Van Golde LM, Verhoef J (1990). Impairment of phagocytic functions of alveolar macrophages by hydrogen peroxide. Am J Phys Lung Cell Mol Phys.

[CR75] Pacht ER, Timerman AP, Lykens MG, Merola AJ (1991). Deficiency of alveolar fluid glutathione in patients with sepsis and the adult respiratory distress syndrome. Chest..

[CR76] Panopalis P, Yazdany J, Gillis JZ, Julian L, Trupin L, Hersh AO (2008). Health care costs and costs associated with changes in work productivity among persons with systemic lupus erythematosus. Arthritis Care Res.

[CR77] Paoli CJ, Reynolds MA, Sinha M, Gitlin M, Crouser E (2018). Epidemiology and costs of sepsis in the United States—an analysis based on timing of diagnosis and severity level. Crit Care Med.

[CR78] Pilon D, Obando C, Ding Z, Voelker J, Muser E, Manceur AM (2019). The economic burden of ulcerative colitis in the United States. Gastroenterology..

[CR79] Pravda J (2005). Radical induction theory of ulcerative colitis. World J Gastroenterol.

[CR80] Pravda J (2014). Metabolic theory of septic shock. World J Crit Care Med.

[CR81] Pravda J (2019). Systemic lupus Erythematosus: pathogenesis at the functional limit of redox homeostasis. Oxidative Med Cell Longev.

[CR82] Pravda J (2019). Can ulcerative colitis be cured?. Discov Med.

[CR83] Pravda J, Weickert MJ, Wruble LD (2019). Novel combination therapy induced histological remission in patients with refractory ulcerative colitis-case series report. J Inflam Bowel Dis Disor.

[CR84] Prescott HC, Angus DC (2018). Enhancing recovery from sepsis: a review. Jama..

[CR85] Pryor WA, Arbour NC, Upham B, Church DF (1992). The inhibitory effect of extracts of cigarette tar on electron transport of mitochondria and submitochondrial particles. Free Radicals Biol Med.

[CR86] Pryorham N, Selby W, Lazarus R, Solomon M (2003). Is smoking an indirect risk factor for the development of ulcerative colitis? An age-and sex-matched case-control study. Eur J Gastroenterol Hepatol.

[CR87] Rao RK, Baker RD, Baker SS, Gupta A, Holycross M (1997). Oxidant-induced disruption of intestinal epithelial barrier function: role of protein tyrosine phosphorylation. Am J Physiol Gastrointest Liver Physiol.

[CR88] Redza-Dutordoir M, Averill-Bates DA (2016). Activation of apoptosis signaling pathways by reactive oxygen species. Biochim Biophys Acta.

[CR89] Rekvig OP (2018). Systemic lupus erythematosus: definitions, contexts, conflicts, Enigmas. Front Immunol.

[CR90] Rhee C, Jones TM, Hamad Y, Pande A, Varon J, O’Brien C (2019). Prevalence, underlying causes, and preventability of sepsis-associated mortality in US acute care hospitals. JAMA Netw Open.

[CR91] Roche M, Rondeau P, Singh NR, Tarnus E, Bourdon E (2008). The antioxidant properties of serum albumin. FEBS Lett.

[CR92] Rubino F (2015). Toxicity of glutathione-binding metals: a review of targets and mechanisms. Toxics..

[CR93] Sands B, Compton C. Case records of the Massachusetts General Hospital. Case # 36–1997: (1997) A 58-year-old man with recurrent ulcerative colitis, bloody diarrhea, and abdominal distention. New Engl J Med. 337:1532–40. 10.1056/NEJM199711203372108 9366586

[CR94] Santhanam S, Venkatraman A, Ramakrishna BS (2007). Impairment of mitochondrial acetoacetyl CoA thiolase activity in the colonic mucosa of patients with ulcerative colitis. GUT..

[CR95] Segerstrom SC, Miller GE (2004). Psychological stress and the human immune system: a meta-analytic study of 30 years of inquiry. Psychol Bull.

[CR96] Shah D, Sah S, Wanchu A, Wu MX, Bhatnagar A (2013). Altered redox state and apoptosis in the pathogenesis of systemic lupus erythematosus. Immunobiology..

[CR97] Sheenan J, Brynjolfsson G (1960). Ulcerative colitis following hydrogen peroxide enema. Lab Investig.

[CR98] Shenep JL, Stokes DC, Hughes WT (1985). Lack of antibacterial activity after intravenous hydrogen peroxide infusion in experimental Escherichia coli sepsis. Infect Immun.

[CR99] Sies H (2014). Role of metabolic H_2_O_2_ generation redox signaling and oxidative stress. J Biol Chem.

[CR100] Sturza A, Popoiu CM, Ionică M, et al. Monoamine oxidase-related vascular oxidative stress in diseases associated with inflammatory burden. Oxid Med Cell Longev. 2019;2019:8954201. 10.1155/2019/8954201. Published 2019 Apr 15. 10.1155/2019/8954201 PMC6501417 31178977

[CR101] Tatsumi T, Kako KJ (1993). Effects of hydrogen peroxide on mitochondrial enzyme function studied in situ in rat heart myocytes. Basic Res Cardiol.

[CR102] Tewthanom K (2008). Correlation of lipid peroxidation and glutathione levels with severity of systemic lupus erythematosus: a pilot study from single center. J Pharm Pharm Sci.

[CR103] Tinsley KW, Grayson MH, Swanson PE, Drewry AM, Chang KC, Karl (2003). Sepsis induces apoptosis and profound depletion of splenic interdigitating and follicular dendritic cells. J Immunol.

[CR104] Tozzi-Ciancarelli MG, Di CM, D'Orazio MC, Mascioli A, Di AG, Tozzi E (1990). Effect of exogenous hydrogen peroxide on human erythrocytes. Cell Mol Biol.

[CR105] Tretter L, Adam-Vizi V (2000). Inhibition of Krebs cycle enzymes by hydrogen peroxide: A key role of [alpha]-ketoglutarate dehydrogenase in limiting NADH production under oxidative stress. J Neurosci.

[CR106] Tretter L, Adam-Vizi V (2004). Generation of reactive oxygen species in the reaction catalyzed by α-ketoglutarate dehydrogenase. J Neurosci.

[CR107] Tretter L, Adam-Vizi V (2005). Alpha-ketoglutarate dehydrogenase: a target and generator of oxidative stress. Philos Trans R Soc Lond B Biol Sci.

[CR108] Tripathi K, Feuerstein JD (2019). New developments in ulcerative colitis: latest evidence on management, treatment, and maintenance. Drugs Context.

[CR109] Tsang RY, Al-Fayea T, Au HJ (2009). Cisplatin overdose: toxicities and management. Drug Saf.

[CR110] Upchurch GR, Welch GN, Fabian AJ, Freedman JE, Johnson JL, Keaney JF (1997). Homocyst(e) ine decreases bioavailable nitric oxide by a mechanism involving glutathione peroxidase. J Biol Chem.

[CR111] US Department of Health and Human Services.n.d. Chemical Hazards emergency medical management. https://chemm.nlm.nih.gov/countermeasure_sodium-thiosulfate.htm#indication. Accessed 20 Mar 2020.

[CR112] van Asbeck BS, Braams R, Aarsman JM, Sprong RC, Groenewegen GA (1995). Hydrogen peroxide in blood of patients with Sepsis syndrome: A realistic phenomenon. Crit Care Med.

[CR113] Viswanathan K, Dhabhar FS (2005). Stress-induced enhancement of leukocyte trafficking into sites of surgery or immune activation. Proc Natl Acad Sci.

[CR114] Weiss SJ (1982). Neutrophil-mediated methemoglobin formation in the erythrocyte. The role of superoxide and hydrogen peroxide. J Biol Chem.

[CR115] Wetter DA, Davis MD (2006). Ulceration of the arm attributed to a spider bite and treated with intravenous hydrogen peroxide: a cautionary tale. Arch Dermatol.

[CR116] Wilcken DE, Wang XL, Adachi T, Hara H, Duarte N, Green K (2000). Relationship between homocysteine and superoxide dismutase in homocysteinuria. Aterioscler Thromb Vasc Biol.

[CR117] Wong HS, Dighe PA, Mezera V, Monternier PA, Brand MD (2017). Production of superoxide and hydrogen peroxide from specific mitochondrial sites under different bioenergetic conditions. J Biol Chem.

[CR118] Xiang J, Wan C, Guo R, Guo D (2016). Is hydrogen peroxide a suitable apoptosis inducer for all cell types?. BioMed Res Int.

[CR119] Yoon KW (2017). Dead cell phagocytosis and innate immune checkpoint. BMB Rep.

[CR120] Zanotti-Cavazzoni SL, Hollenberg SM (2009). Cardiac dysfunction in severe sepsis and septic shock. Curr Opin Crit Care.

[CR121] Zhong Y, Yan F, Jie W, Zhou Y, Fang F (2019). Correlation between serum homocysteine level and ulcerative colitis: A meta-analysis. Pteridines..

[CR122] Zorov DB, Juhaszova M, Sollott SJ (2006). Mitochondrial ROS-induced ROS release: an update and review. Biochim Biophys Acta.

